# Lenalidomide Combined with Interferon α-1b and Interleukin-2 in the Treatment of 21 Cases of Acute Myeloid Leukemia

**DOI:** 10.4274/tjh.galenos.2021.2020.0050

**Published:** 2021-08-25

**Authors:** Cheng Cheng, Ruihua Mi, Dongbei Li, Lin Chen, Xudong Wei

**Affiliations:** 1 Affiliated Tumor Hospital of Zhengzhou University, Henan Cancer Hospital, Department of Hematology, Zhengzhou, China

**Keywords:** Acute myeloid leukemia, Refractory/relapsed, Minimal residual disease, Lenalidomide, Interferon α-1b, Interleukin-2

## To the Editor,

The prognosis of patients with refractory/relapsed acute myeloid leukemia (R/R AML) is extremely poor and the long-term survival rate is less than 10%. Minimal residual disease (MRD) is an important independent prognostic indicator of AML, indicating a higher risk of recurrence; thus, it is vital for the prognosis of patients to eliminate MRD [1,2]. Our center previously used thalidomide combined with interferon α-1b (IFN α-1b) and interleukin-2 (IL-2) in the treatment of R/R AML and the total effective rate was 50% [3,4]. We further optimized the treatment plan, adjusted thalidomide to lenalidomide, and applied it for 21 patients with R/R AML or MRD.

All patients were treated with lenalidomide combined with the IFN α-1b and IL-2 regimen. The specific treatment plan was as follows: oral administration of lenalidomide capsule, 10-25 mg, every night; IFN-α1b, 60 µg; and IL-2, 1,000,000 U subcutaneous injection, once every other day. Each treatment cycle lasted 4 weeks. This retrospective analysis was approved by the Institutional Review Board of Henan Cancer Hospital.

Among 17 patients with R/R AML, 7 patients had complete remission (CR), 2 had CR with incomplete recovery of blood cells (CRi), and 8 had no remission. One patient in the low-risk group achieved CR, while the remission rate in the intermediate-risk group and high-risk group was 57.1% (4/8) and 50% (4/8), respectively. Of 3 patients with *TET2* mutations, 2 patients achieved remission; 6 patients with *FLT3-ITD/TKD *mutations were given sorafenib at the same time and 3 patients achieved remission. Particularly, among the 4 MRD-positive patients with remission of AML, the MRD of 3 patients was lower than before and the MRD of 1 patient was higher than before. These patients’ clinical data are presented in Table 1. The total effective rate (CR+CRi+MRD decreased) of 21 patients was 57.1%. No treatment-related deaths occurred. The median overall survival time of the 21 patients was 26 months (9-89 months), and the 3-year survival rate reached 42.9%. Among patients with effective application of this regimen, the duration of relief ranged from 2 to 28+ months.

IFN can directly kill AML by inhibiting growth-promoting cytokines, inducing apoptosis, and inhibiting cell proliferation. Meanwhile, it is possible to indirectly target AML cells through the immunostimulatory effects of interferon on dendritic cells, T-cells, and natural killer (NK) cells [5,6]. IL-2 increases the proliferation and activity of cytotoxic T-cells, NK-cells, and killer cells activated by lymphokines. It can also promote the secretion of antibodies and interferon to play an anti-tumor role [7]. Lenalidomide promotes tumor cell apoptosis by inhibiting the secretion of tumor necrosis factor α, IL-1, and IL-12. It also produces an anti-tumor effect by inhibiting the secretion of overexpressed vascular endothelial growth factor [8,9,10].

The combination of lenalidomide, IFN α-1b, and IL-2 showed improvements in efficacy and safety profiles as compared to monotherapy among patients with R/R AML, and especially in the elimination of MRD, and it may become a promising treatment regimen.

## Figures and Tables

**Table 1:**
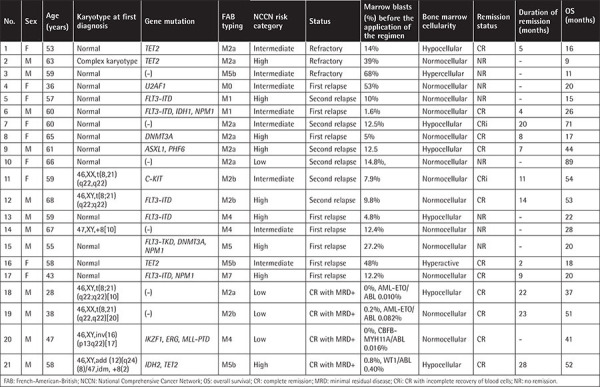
Clinical data of 21 patients with refractory/relapsed or minimal residual disease-positive acute myeloid leukemia treated with lenalidomide combined with interferon α-1b and interleukin-2.
